# Supplementary material for: Functional annotation of enzyme-encoding genes using deep learning with transformer layers
Source: Nat Commun. 2023 Nov 14;14:7370. doi: 10.1038/s41467-023-43216-z (PMC10645960; doi:10.1038/s41467-023-43216-z)
Supplement: Supplementary file 1 — Supplementary Information [file 41467_2023_43216_MOESM1_ESM.pdf]

Supplementary Information for

Functional annotation of enzyme-encoding genes using deep learning with transformer layers

Kim et al.

Supplementary Notes

Supplementary Note 1. Experimental validation of malate dehydrogenase activity of P93052 *in vivo*

To validate the prediction, we expressed the enzyme P93052 in *Mannheimia succiniciproducens*, a facultative anaerobic rumen bacterium that naturally produces succinic acid[1]. In a previous study, the pathways producing byproducts of *M. succiniciproducens* were removed to improve succinic acid production by deleting L-lactate dehydrogenase, phosphate acetyltransferase, and acetate kinase, that are responsible for the production of lactic and acetic acids[2]. The fed-batch fermentation of the resulting PALK strain produced 74.56 g l^{-1} of succinic acid without lactic acid accumulation throughout the fermentation, when glucose was used as the carbon source[3]. To investigate whether P93052 is L-lactate dehydrogenase or malate dehydrogenase, we constructed the PALK strain, which overexpressed P93052. The fed-batch fermentation of the resulting strain PALK (pMS3-P93052) produced 86.52 g l^{-1} of succinic acid without lactic acid accumulation using glucose as a carbon source (Supplementary Fig. 12). These results suggest that P93052 functions as a malate dehydrogenase (EC:1.1.1.37). This was further supported by previous studies, which have demonstrated that overexpression of malate dehydrogenase from different microorganisms led to improved succinic acid production[4]. The detailed description of the experiment is described in Supplementary Note 4.

Supplementary Note 2. Interpretation of DeepECtransformer predictions for the enzymes with mis-annotated EC numbers

DeepECtransformer predicted the EC numbers that are different from the annotations in UniProtKB/Swiss-Prot. To understand these predictions, we examined the attention scores computed in the self-attention layers. For instance, P93052, which was annotated as an L-lactate dehydrogenase (EC:1.1.1.27), was predicted as malate dehydrogenase (EC:1.1.1.37) by DeepECtransformer with the neural network prediction score of 0.9703. Upon further investigation, we identified a protein A0A147JD58_9SPHN, which was in the training dataset of the DeepECtransformer neural network, with the same EC number (EC:1.1.1.37) and a high sequence identity (96.6%). To confirm whether the prediction was solely made by the high sequence identity, motifs assigned with high attention scores were analyzed (Supplementary Fig. 14). We found that the highlighted motifs were shown in TIGR01763, an NCBIfam family associated with malate dehydrogenase. This demonstrates that DeepECtransformer not only relies on sequence similarity but also identifies motifs essential for the enzyme functionality. Similarly, we analyzed how DeepECtransformer predicted the enzyme function of Q038Z3 as dihydroorotate dehydrogenase (NAD) (EC:1.3.1.14), which was previously annotated as dihydroorotate dehydrogenase (fumarate) (EC:1.3.98.1). DeepECtransformer neural network predicted Q038Z3 as dihydroorotate dehydrogenase (NAD) with the prediction score of 0.9791. Although dihydroorotate dehydrogenase (NAD) sequence that had the highest sequence identity was A0A508Z5W5_LACRH, of which sequence identity being 86.6%, the protein sequence in the training dataset that had the highest sequence identity was S2S6H3_LACPA (99.6%), which was annotated as dihydroorotate dehydrogenase (fumarate). When the motifs assigned with high attention scores were analyzed, conserved residues of cd04740, a CDD domain for

DHOD_1B_like domain that represents the dihydroorotate dehydrogenase class 1B FMN-binding domain, were observed (Supplementary Fig. 15). We further analyzed the rationale of DeepECtransformer predictions for Q9WVK7 and C9K7D8. Even though the highest sequence identity with Q9WVK7 and NADP-dependent 3-hydroxybutyryl-CoA dehydrogenase sequences in the training dataset was 51.7% (A0A518EY01_9BACT), DeepECtransformer predicted EC numbers of Q9WVK7 as EC:1.1.1.157 (NADP-dependent 3-hydroxybutyryl-CoA dehydrogenase) with the prediction score of 0.6765, along with the annotated EC number (EC:1.1.1.35; 3-hydroxyacyl-CoA dehydrogenase). When the motifs with high attention scores were analyzed, a motif for NADP-binding proteins, [VILF]-X-G-X-[GSA]-X_2-[GAS]-X_6-[LAIFWCG], was observed (Supplementary Fig. 16)[5]. For C9K7D8, which has the highest sequence similarity of 49.4% (Q4WKQ2_ASPFU; EC:2.6.1.42) with the protein sequences in the training dataset, DeepECtransformer predicted the protein as a branched-chain amino acid transaminase (EC:2.6.1.42) with the prediction score of 0.9711. The motifs with high attention scores were shown in PTHR42825, a PANTHER family for amino acid aminotransferase (Supplementary Fig. 17). The EC number for Q8U4R3 was predicted as EC:4.4.1.5 (a D-cysteine desulfhydrase) with a prediction score of 0.8477, which was originally annotated as 1-aminocyclopropane-1-carboxylaste deaminase (EC:3.5.99.7). The protein sequence in the training dataset that has the highest sequence identity with Q8U4R3 was A0A162MS80_9FIRM (EC:4.4.1.15), having a sequence identity of 51.1%. Because the metabolic reaction of 1-aminocyclopropane-1-carboxylaste deaminase is a sub-reaction of the metabolic reaction of D-cysteine desulfhydrase, we were unable to identify motifs with high attention scores that specifically represent D-cysteine desulfhydrase, rather than 1-aminocyclopropane-1-carboxylaste deaminase.

Supplementary Note 3. Comparative performance of EC number prediction tools

To evaluate the performance of DeepECtransformer, CLEAN, HDMLF, and ProteInfer, we conducted a comparative analysis using Price-149 dataset and NEW-392 dataset provided by Yu et al. (Supplementary Table 2 and 3)[6]. Among the evaluated tools, CLEAN showed the highest performance on the Price-149 dataset, achieving an F_1 score of 0.4947, while DeepECtransformer followed with an F_1 score of 0.3511. While BLASTp exhibited comparable prediction performance to deep learning-based EC number prediction tools, the utilization of graphics processing unit (GPU)-based acceleration allows deep learning-based tools to achieve high-throughput prediction of EC numbers. CLEAN also showed the highest performance of the NEW-392 dataset, achieving an F_1 score of 0.4967, followed by DeepECtransformer (0.3350) and ProteInfer (0.3341). The Price-149 dataset comprises 149 amino acid sequences covering only 56 types of EC numbers and the NEW-392 dataset comprises 392 amino acid sequences covering 177 types of EC numbers. To evaluate the prediction performance on a larger benchmarking dataset, we compared the performance of CLEAN and DeepECtransformer, both of which offer high-throughput prediction APIs. For this analysis, we constructed the SwissProt2023 dataset by extracting 1,239 amino acid sequences that cover 480 types of EC numbers from SwissProtKB, which were published since April 2022. Upon evaluating the prediction results of CLEAN and DeepECtransformer on this dataset, CLEAN achieved an F_1 score of 0.2530, whereas DeepECtransformer achieved an F_1 score of 0.2889 (Supplementary Table 4). The evaluated metrics in Supplementary Table 2, 3 and 4 were calculated using weighted metrics in scikit-learn v0.21.3.

In addition, we evaluated the ability of ProteInfer, HDMLF, and CLEAN to predict the EC numbers of YgfF, YciO, and YjdM, of which EC numbers were predicted by DeepECtransformer and validated through *in vitro* enzyme assays. ProteInfer and HDMLF failed to predict any EC

numbers for YgfF, YciO, and YjdM. On the other hand, CLEAN predicted EC numbers for YciO (EC:2.7.7.87) as EC:2.7.7.87 and EC:3.1.26.5. However, CLEAN failed to correctly predict the EC numbers for YgfF and YjdM. CLEAN predicted YgfF as EC:1.1.1.138 and YjdM as EC:2.7.7.6, instead of their correct EC numbers (EC:1.1.1.47 and EC:3.11.1.2, respectively).

Supplementary Note 4. Materials and methods for the fed-batch fermentation of PALK (pMS3-P93052)

The strains, plasmids, and oligonucleotides used in this study are listed in Supplementary Table 5. To construct pMS3-P93052, pMS3 was with linearized using *Eco*RI and *Kpn*I. *P93052* gene fragment was prepared by polymerase chain reaction using the primers P1/2 and the synthesized gene fragment *P93052* as a template. Prepared *P93052* gene fragment was then ligated with linearized pMS3 using Gibson assembly. Correct vector construction was verified using DNA sequencing. pMS3-P93052 was then transformed to PALK strain resulting in PALK (pMS3-P93052) strain following the procedures described in previous reports[3, 4].

M. succiniciproducens strains were precultured in a 500 ml Erlenmeyer flask equipped with gas inlet and outlet ports. Each flask contained 250 ml of complex medium (per l: 5 g yeast extract, 1 g NaCl, 0.02 g $CaCl_2{\cdot}2H_2O$, 0.2 g $MgCl_2{\cdot}6H_2O$, and 8.709 g K_2HPO_4). After adjusting the pH of the medium to 7.0 by the addition of 5 N NaOH and flushing with CO_2, it was heat sterilized at 121 ºC for 15 min. A separately heat-sterilized carbon source, glucose and/or glycerol were added into the medium before inoculating 2.5 ml of glycerol stock culture (15%, w/v) stored in a deep freezer at -70 ºC. Flasks charged with CO_2 as a headspace gas were incubated in a static incubator (JEIO TECH, Daejeon, Korea) at 39 ºC.

Batch and fed-batch fermentations were carried out in a 6.6 L Bioflo 3000 bioreactor (New Brunswick Scientific Co., Edison, NJ, USA) with a working volume of 2.5 L. The composition of the CDM used in this study contained (per L) 1 g NaC1, 0.02 g $CaCl_2{\cdot}2H_2O$, 2 g $(NH_4)_2SO_4$, 0.5 g alanine, 0.5 g asparagine, 0.005 g biotin, 0.5 g methionine, 0.005 g Ca-pantothenate, 0.005 g

pyridoxine-HCl, 0.005 g thiamine, 0.2 g $MgCl_2 \cdot 6H_2O$, 1.5 g $K_2H_2PO_4$, 9.997 g $NaHCO_3$, 0.005 g ascorbic acid, 0.5 g aspartic acid, 0.5 g cysteine, 0.005 g nicotinic acid, 0.5 g proline, 0.5 g serine, and 5 ml trace metal solution. The trace metal solution contained (per L) 5 ml HCl, 10 g $FeSO_4 \cdot 7H_2O$, 2.25 g $ZnSO_4 \cdot 7H_2O$, 1 g $CuSO_4 \cdot 5H_2O$, 0.5 g $MnSO_4 \cdot 5H_2O$, 0.23 g $NaB_4O_7 \cdot 10H_2O$, and 0.1 g $(NH_4)_6MO_7O_{24}$. The CDM was supplemented with 18.02 g l^{-1} (100 mM) glucose. Antibiotics were added to the following concentration when necessary: Ap (25 mg l^{-1}) and Km (25 mg l^{-1}). The feeding solution was composed of 900 g l^{-1} of glucose as indicated. Fed-batch fermentation was initiated by inoculation of 300 ml of precultured broth, giving the initial optical density (OD_{600}) of 0.2-0.3. Temperature and agitation speed of four flat blade turbine impellers in the bioreactor were controlled at 39 °C and 200 rpm, respectively. The pH of the fermentation broth was controlled at 6.5 by automatic addition of a mixture of 1.57 M of ammonia solution and 6.84 M of $MgOH_2$ solution. The bioreactor was continuously sparged with industrial-grade CO_2 gas at a flow rate of 0.2 vvm (CO_2volume per working volume per min) by a mass flow controller. Fed-batch fermentations were performed in a semi-continuous feeding mode, in which the feeding solution was supplied into the bioreactor via a peristaltic pump, to maintain the carbon source concentration at 5-15 g l^{-1} by changing the feeding rate to minimize substrate inhibition.

The concentrations of glucose and fermentative products were immediately monitored using ProStar 210 HPLC (Varian, CA, USA) accompanied with ProStar 320 UV/visible-light (Varian) and Shodex RI-71 refractive index (Shodex, Tokyo, Japan) detectors over the entire period of fermentation. The MetaCarb 87H column (300 × 7.8 mm; Agilent, CA, USA) was eluted isocratically (flow rate = 0.6 ml min^{-1}) using 0.01 N H_2SO_4 at 60 °C. Cell growth was monitored by measuring OD_{600} using Ultrospec 3000 spectrophotometer (GE Healthcare, Chalfont St. Giles, UK).

Supplementary Figures

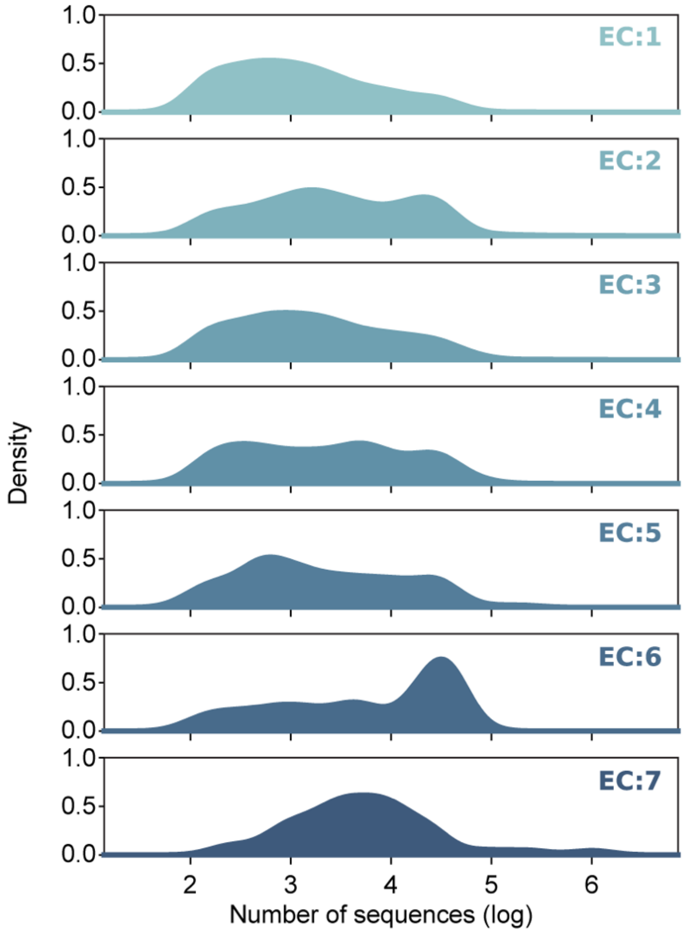

Supplementary Fig. 1. The density plots of the distribution of the number of sequences for EC number classes in the uniprot dataset. Each individual plot represents the density of the number of sequences for EC numbers that have the same first-level EC number.

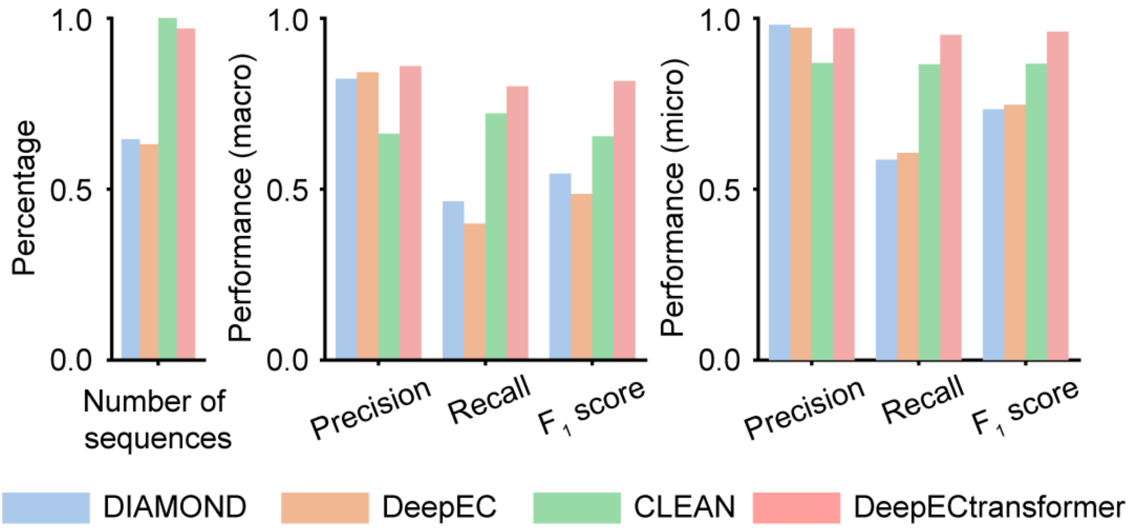

Supplementary Fig. 2. Comparison of the prediction performance of EC number prediction tools, DIAMOND, DeepEC, CLEAN, and DeepECtransformer. The performance tests were performed for the test dataset.

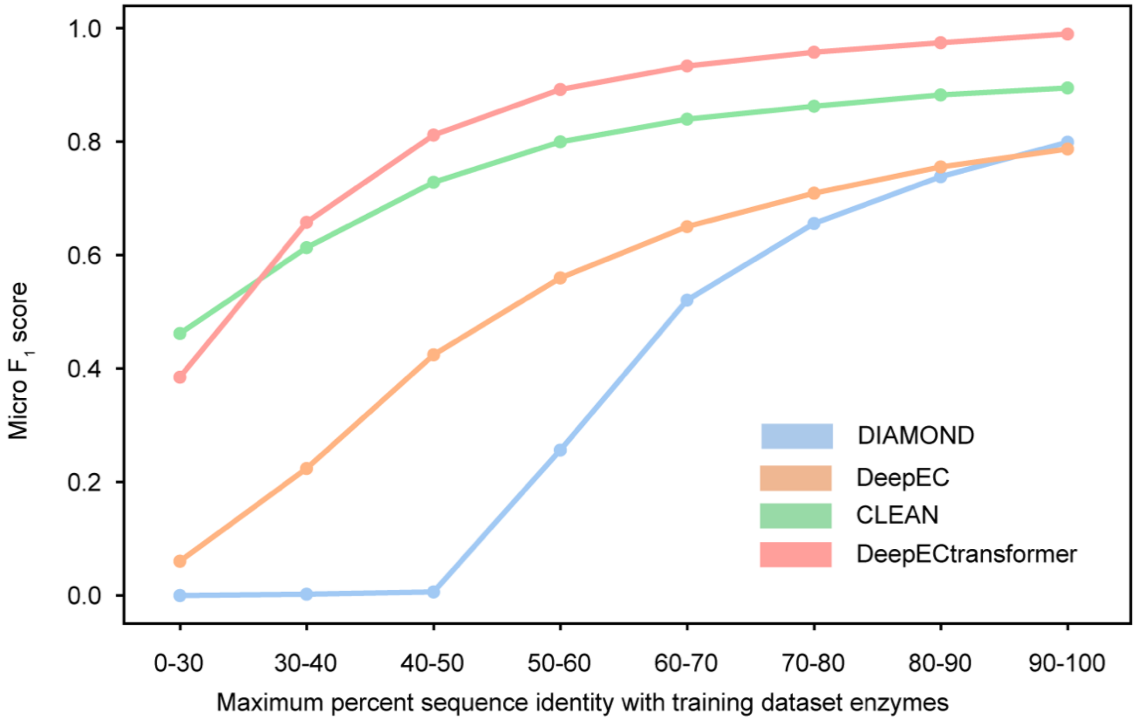

Supplementary Fig. 3. Prediction performance of the EC number prediction tools per maximum sequence identity with training dataset. The maximum sequence identities of enzymes in the test dataset were calculated using DIAMOND, aligning the amino acid sequences of enzymes in the test dataset as the query sequences to the amino acid sequences of enzymes in the training dataset as the reference sequences.

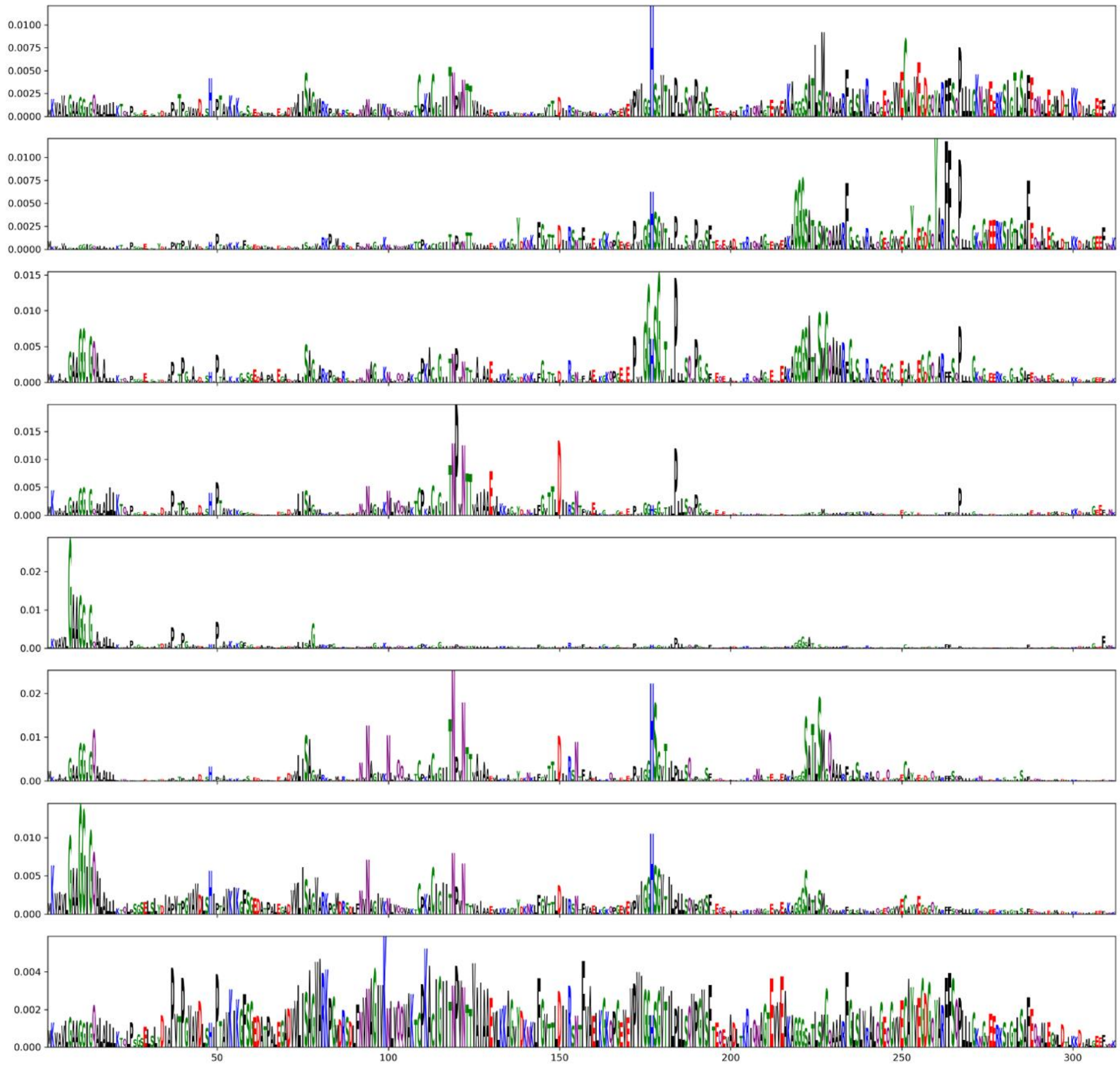

Supplementary Fig. 4. Attention scores of NAD-dependent malate dehydrogenase of *E. coli* K-12 for attention heads in the second self-attention layer of DeepECtransformer neural network.

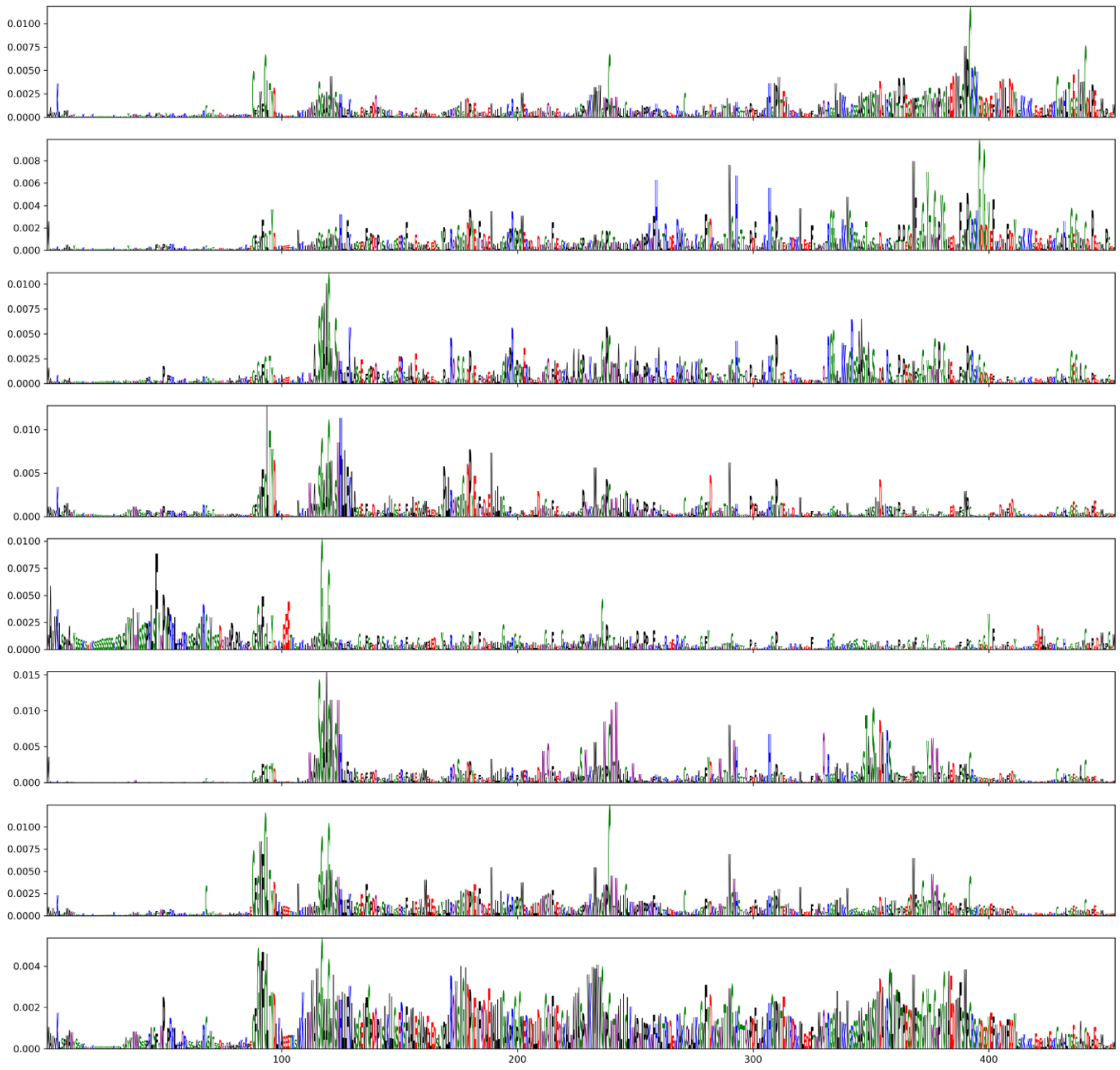

Supplementary Fig. 5. Attention scores of NADP-dependent malate dehydrogenase of *F. bidentis* for attention heads in the second self-attention layer of DeepECtransformer neural network.

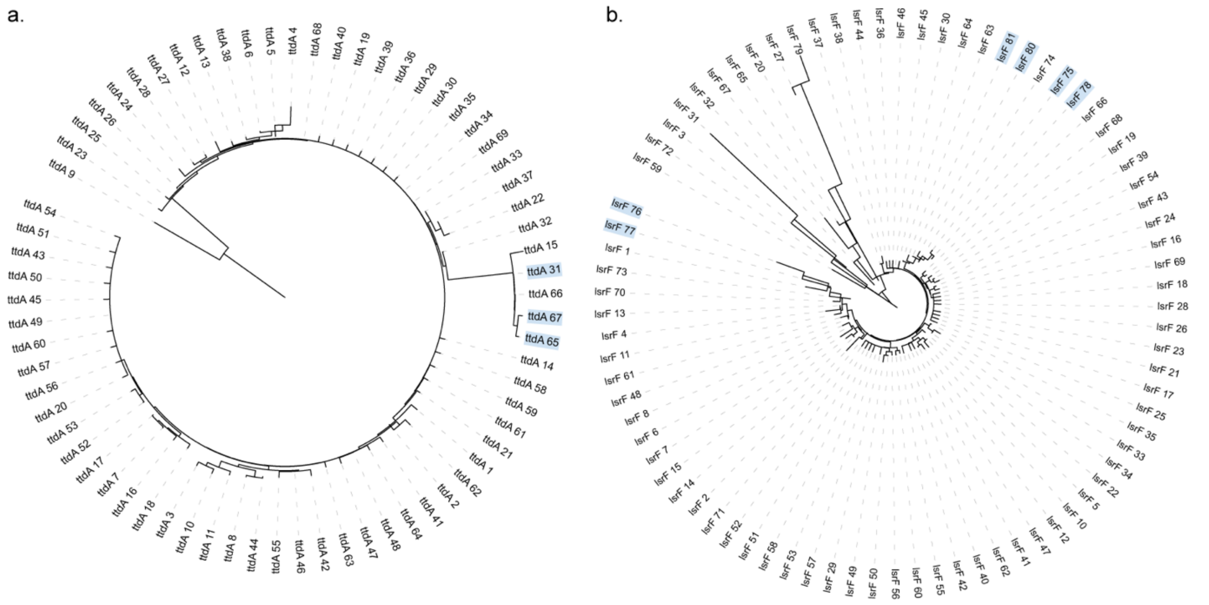

Supplementary Fig. 6. Phylogenetic trees of **a.** *ttdA* and **b.** *lsrF* alleles in 1,122 *E. coli* strains.

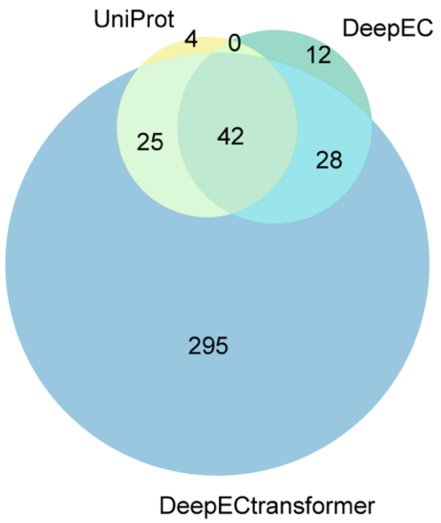

Supplementary Fig. 7. Venn diagram of the EC number of the y-ome proteins. EC numbers with all four digits are analyzed

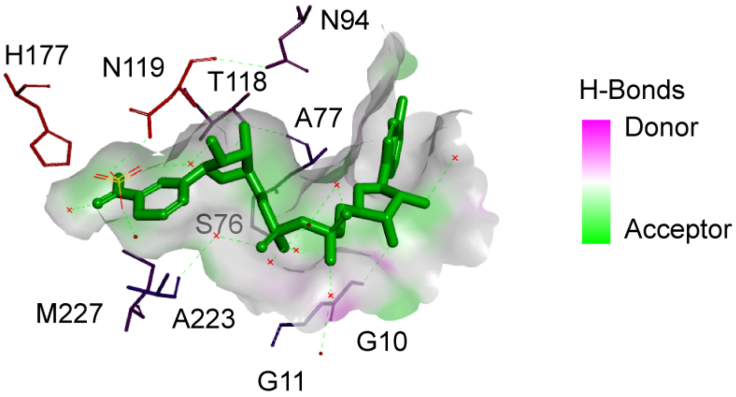

Supplementary Fig. 8. Highlighted amino acid residues by DeepECtransformer neural network for the prediction of EC number of NAD-dependent malate dehydrogenase of *E. coli* K-12 (Protein Data Bank [PDB] ID code 1IB6 [[https://www.rcsb.org/structure/1IB6]).

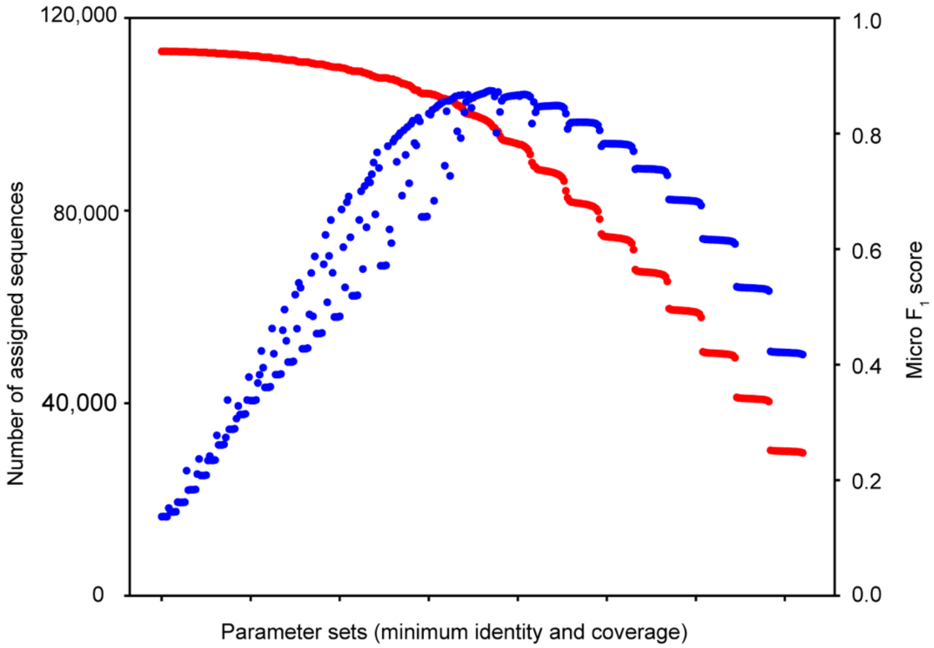

Supplementary Fig. 9. Hyperparameter optimization of the homologous enzyme search using DIAMOND. The minimum percent of sequence identity and coverage were optimized by searching 361 parameter sets (parameter sets on the x-axis). Red and blue dots represent the micro F_1 score of the parameter set and the number of EC number assigned sequences by the homologous search, respectively. A parameter set with a minimum sequence identity of 50% and a minimum sequence coverage of 75% showed the highest micro F_1 score was selected as the hyperparameter set used in this study.

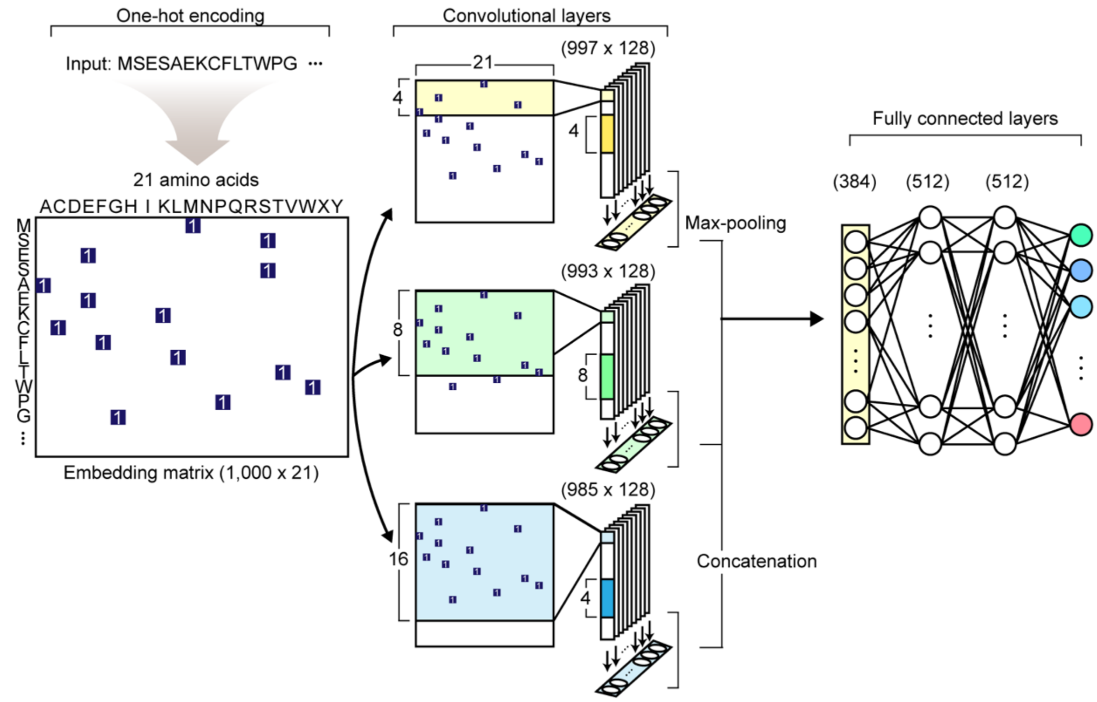

Supplementary Fig 10. Network architecture of DeepEC.

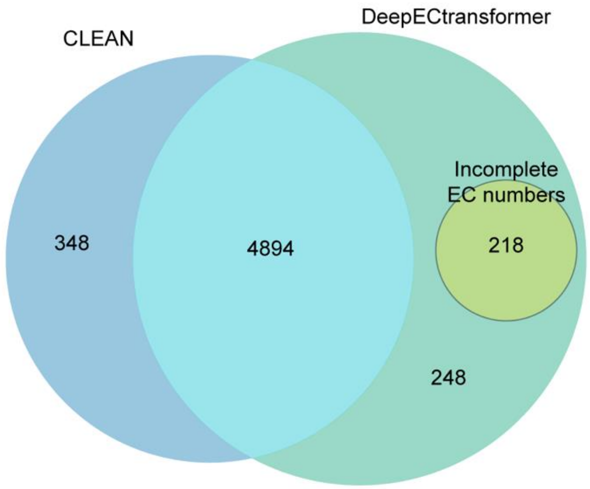

Supplementary Fig 11. Venn diagram of the EC number coverage of DeepECtransformer and CLEAN.

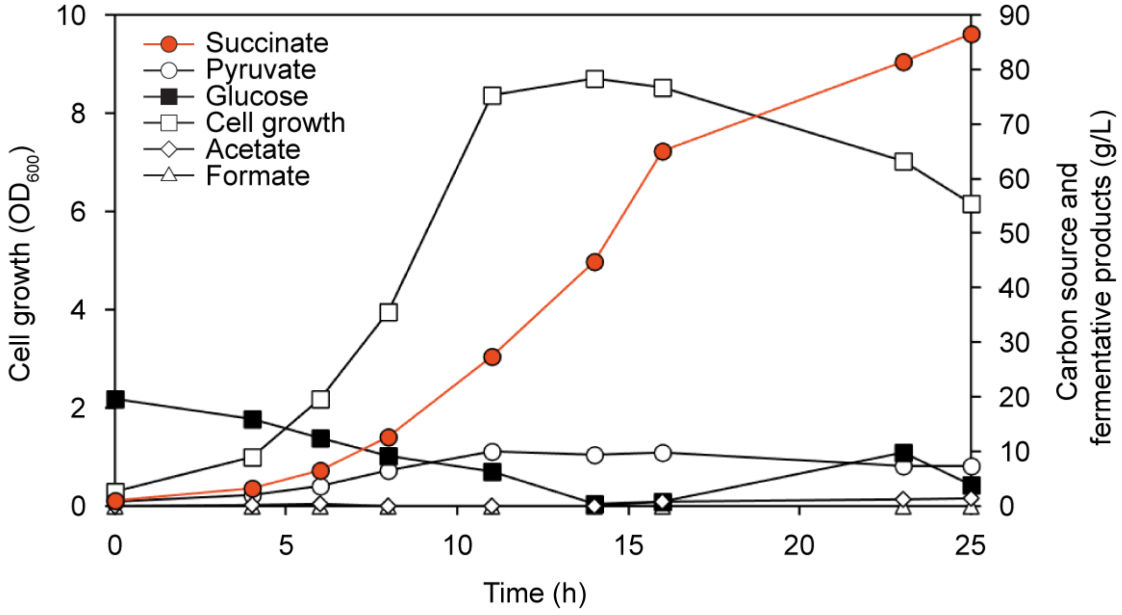

Supplementary Fig 12. Fed-batch fermentation profiles of the *M. succiniciproducens* PALK (pMS3-P93052) strain in chemically defined medium using glucose as carbon source. Symbols: white square, cell growth; red circle, succinic acid; black square, glucose; white circle, pyruvate; white diamond, acetate.

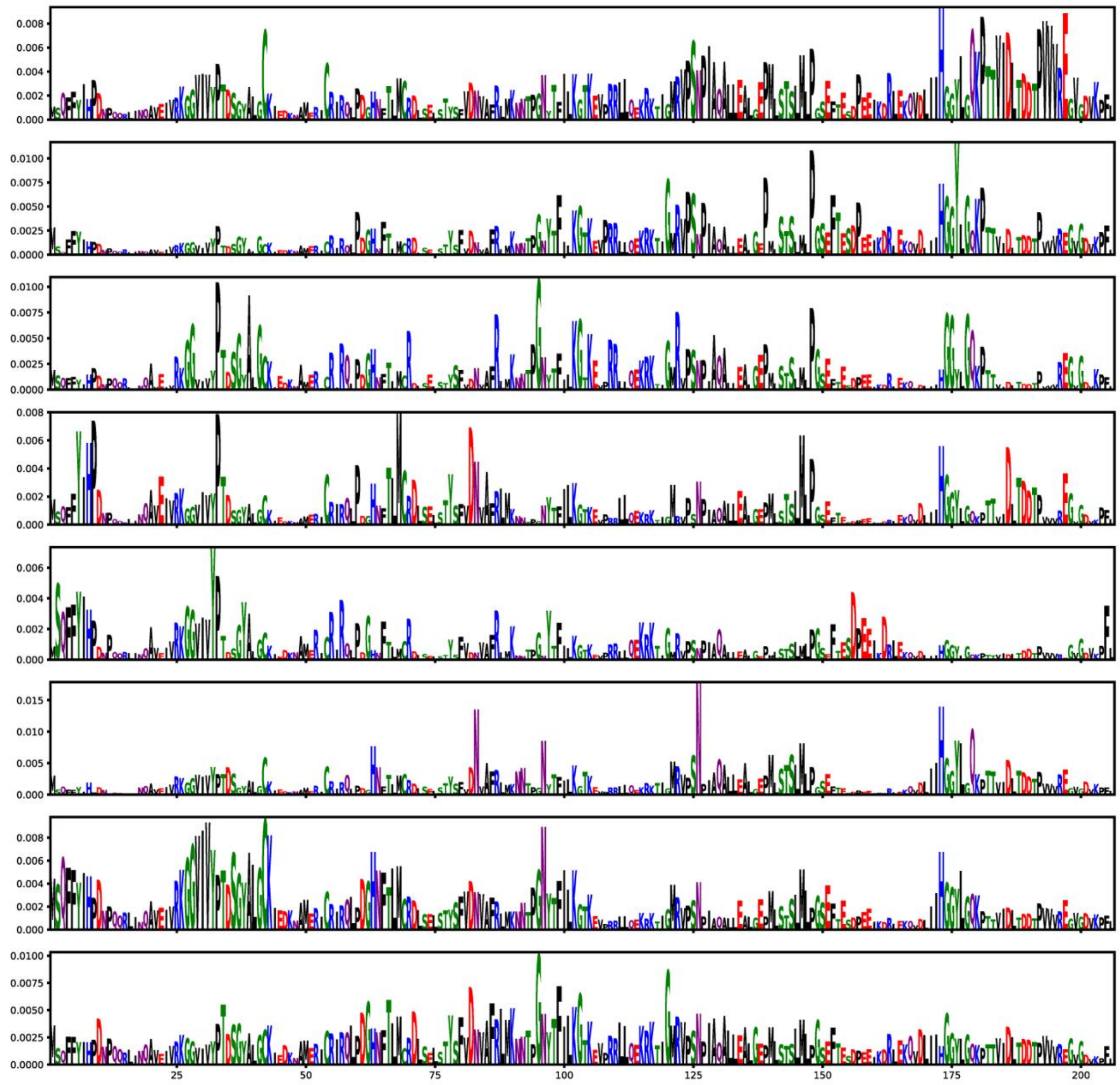

Supplementary Fig 13. Highlighted amino acid residues in DeepECtransformer neural network for EC number prediction of YciO.

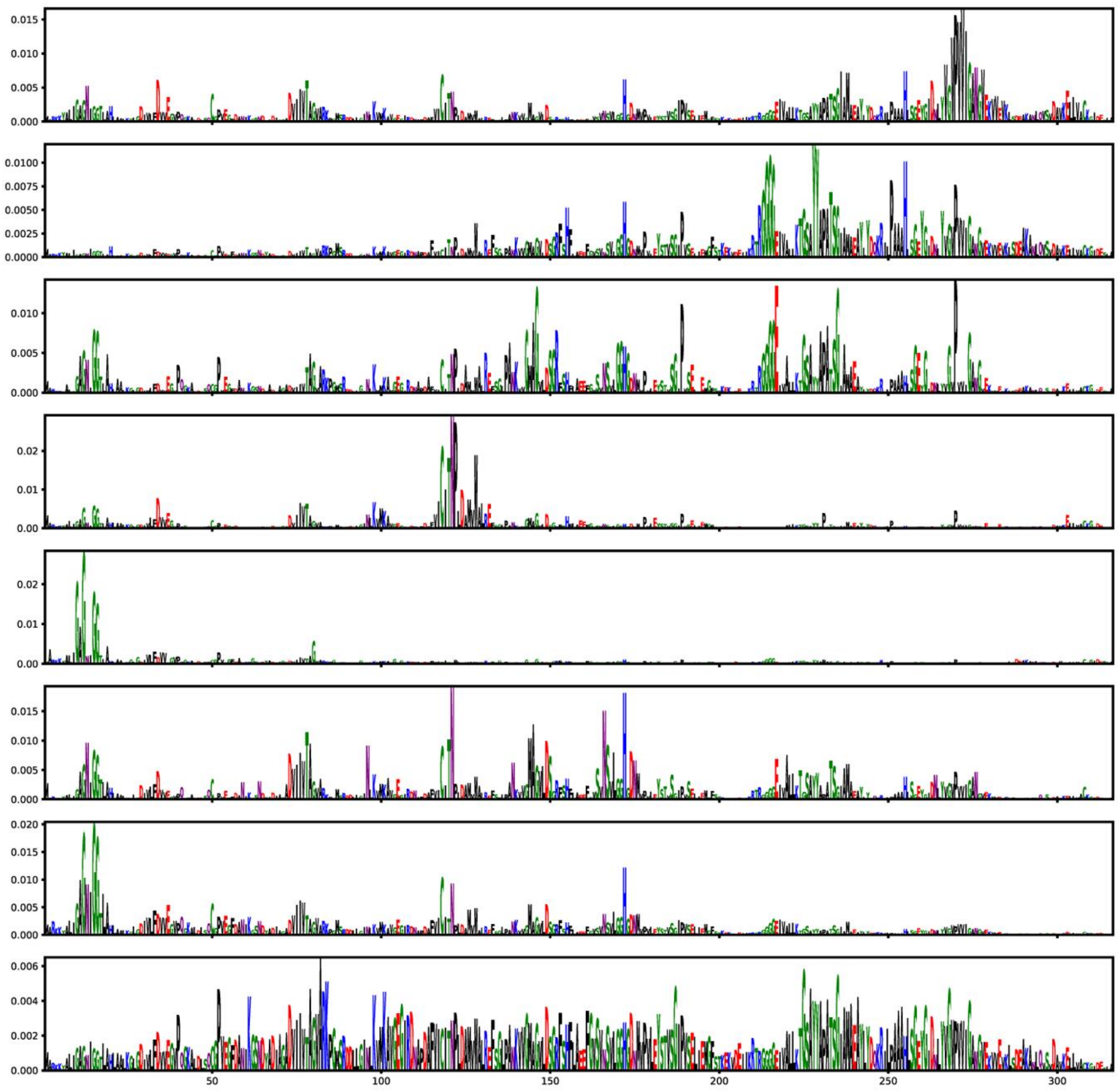

Supplementary Fig 14. Highlighted amino acid residues in DeepECtransformer neural network for EC number prediction of P93052.

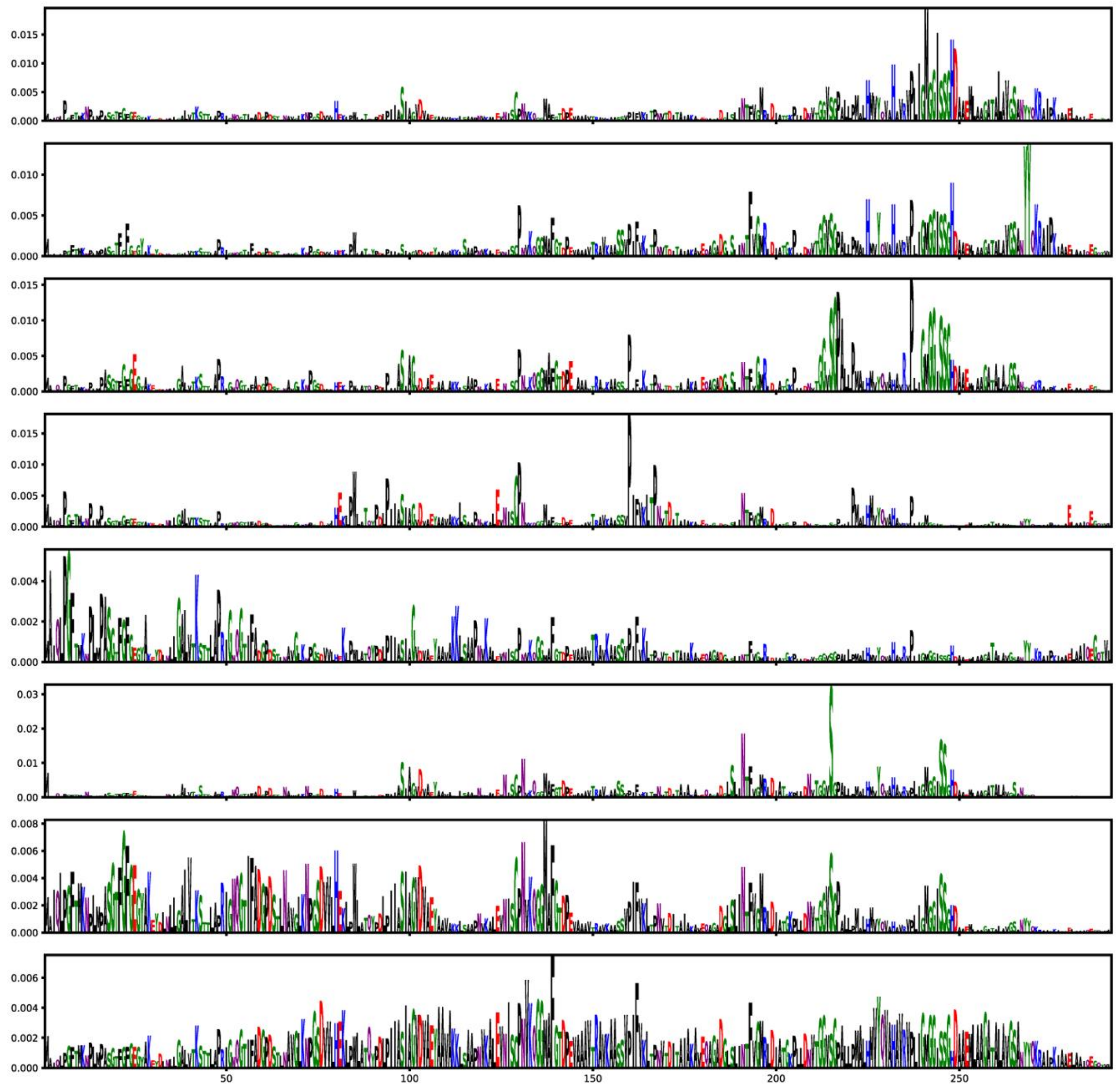

Supplementary Fig 15. Highlighted amino acid residues in DeepECtransformer neural network for EC number prediction of Q038Z3.

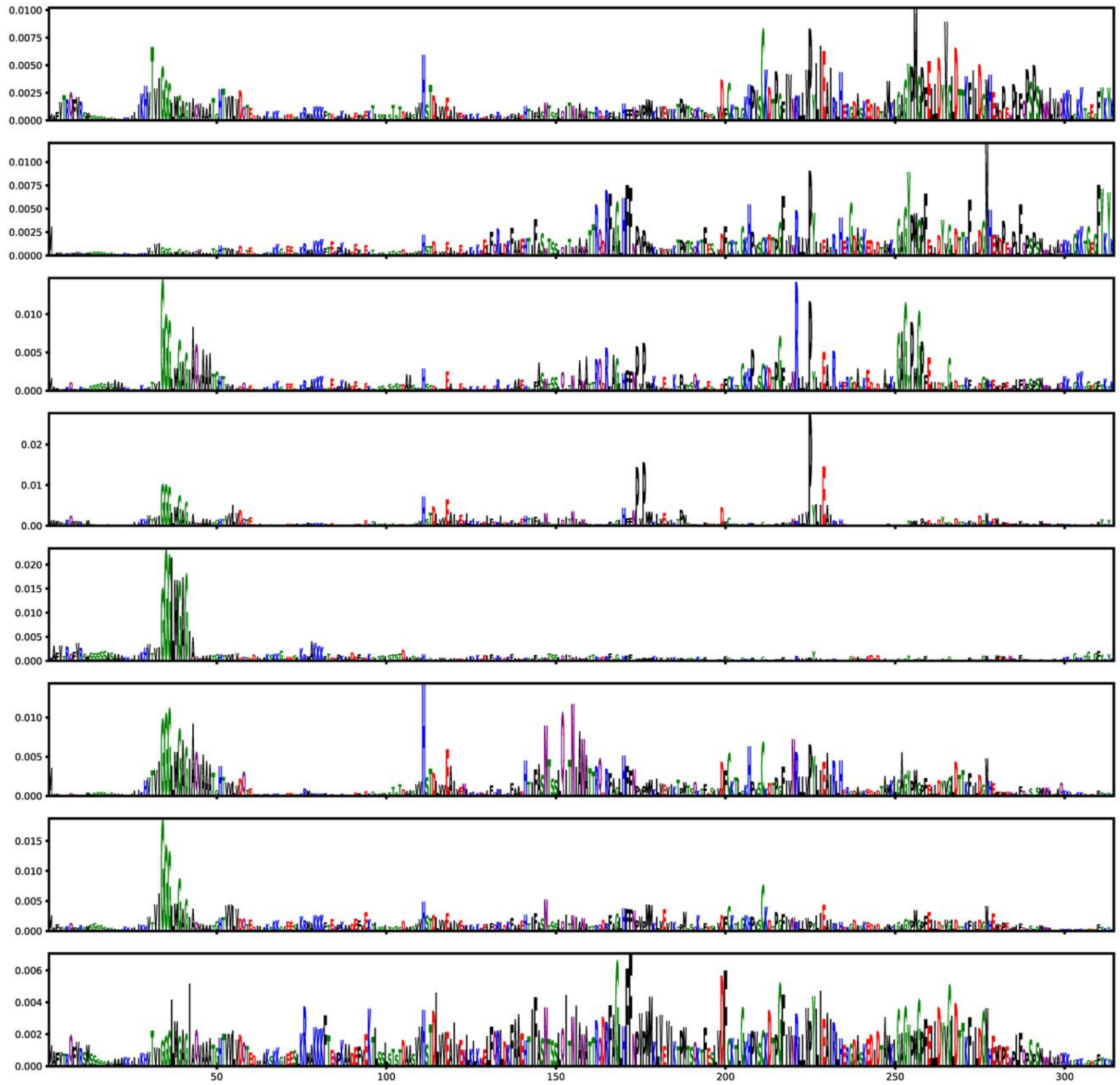

Supplementary Fig 16. Highlighted amino acid residues in DeepECtransformer neural network for EC number prediction of Q9WVK7.

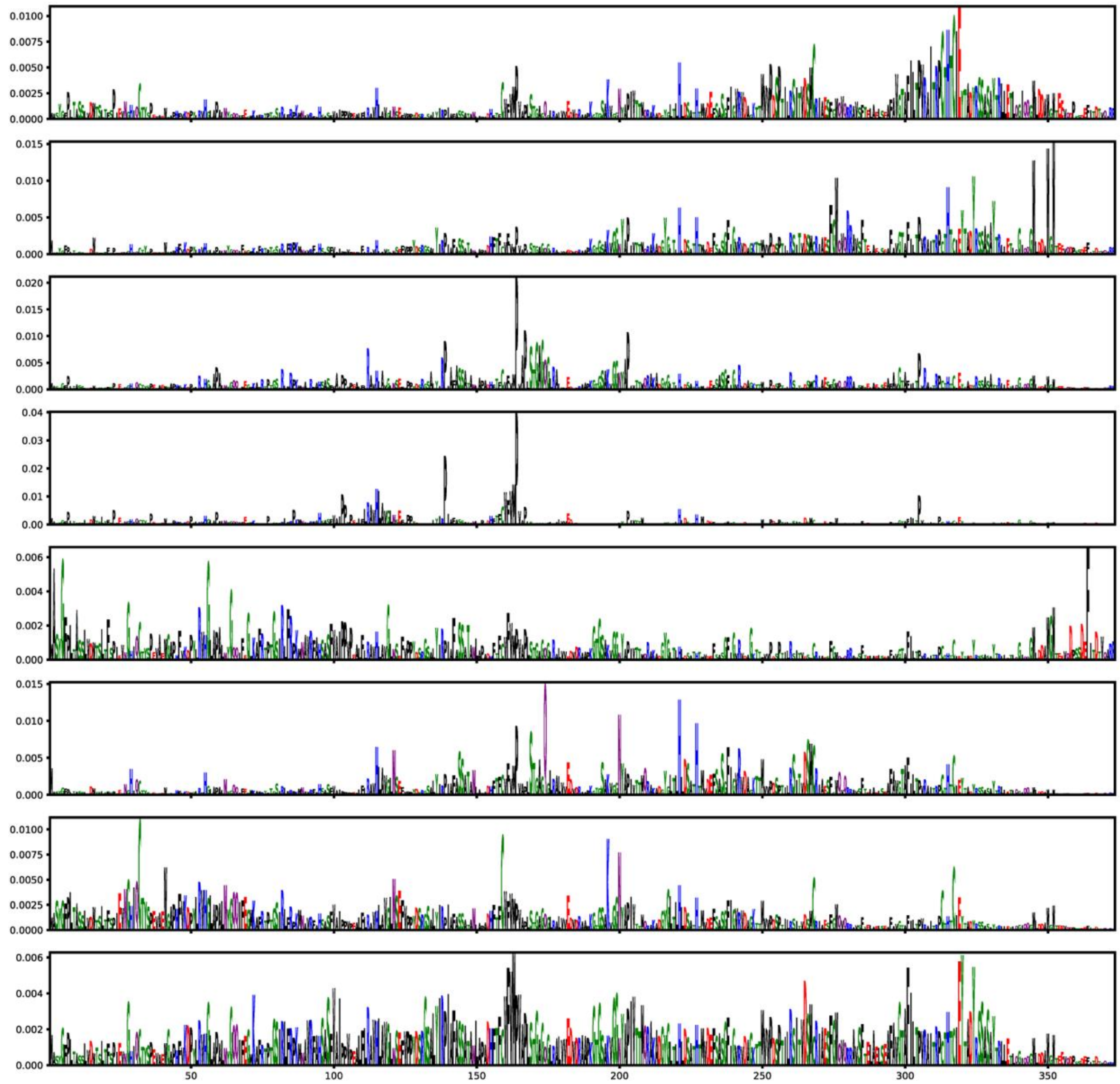

Supplementary Fig 17. Highlighted amino acid residues in DeepECtransformer neural network for EC number prediction of C9K7D8.

Supplementary Tables

Supplementary Table 1. *In vitro* enzyme assay results for YgfF, YciO, and YjdM.

YgfF[a]	1	2	3	Average
Sample (A_0)[b]	0.1179	0.1341	0.1309	0.1276
Sample (A_0, No substrate)[c]	0.0981	0.1068	0.1065	0.1038
Difference between sample with/without substrate ($A_{0,f}$)[d]	0.0198	0.0273	0.0244	0.0238
Sample (A_1)[b]	0.284	0.3114	0.3123	0.3026
Sample (A_1, No substrate)[c]	0.1887	0.2075	0.2069	0.201
Difference between sample with/without substrate ($A_{1,f}$)[d]	0.0953	0.1039	0.1054	0.1016
Difference ($A_{1,f}$-$A_{0,f}$)[d]	0.0755	0.0766	0.081	0.0777
NADH concentration (μM)[e]	1.343	1.3575	1.4155	1.372
YgfF activity (U/mL)[f]	0.8953	0.905	0.9437	0.9147
YgfF concentration (mg/mL)[g]	2.9935	2.9935	2.9935	2.9935
Specific YgfF activity (U/mg)[h]	299.0813	302.3217	315.2352	305.5461

YciO[a]	1	2	3	Average
Sample[b]	3.1198	2.8286	2.7699	2.9061
Sample (No substrate)[c]	2.4126	2.466	2.4407	2.4398
Difference[d]	0.7072	0.3626	0.3292	0.4663
PP_i concentration (nM)[e]	4.433	2.2022	1.9856	2.8736
YciO activity (U/mL)[f]	0.0148	0.0073	0.0066	0.0096
YciO concentration (mg/mL)[g]	0.1852	0.1061	0.1061	0.1325
Specific YciO activity (U/mg)[h]	0.0798	0.0692	0.0624	0.0705

YjdM[a]	1	2	3	Average
Sample[b]	0.5894	0.6105	0.56	0.5866
Sample (No substrate)[c]	0.4189	0.3629	0.4103	0.3974
Difference[d]	0.1705	0.2476	0.1497	0.1893
Phosphate concentration (μM)[e]	99.8821	80.0114	60.1548	80.0161
YjdM activity (U/mL)[f]	166.4702	133.3523	100.258	133.3602
YjdM concentration (mg/mL)[g]	1.206	0.8298	0.8298	0.9552

Specific YjdM activity (U/mg)[h]	138.0368	160.7041	120.8219	139.8543

[a]Before measurement, the standard curves are generated using curve fitting method: NADH standards (ranging from 0 to 10 nmol), PPi standards (ranging from 0 to 1 nmol/well), phosphate standards (ranging from 0 to 40 μM).
[b]Absorbance from sample with its reaction mix.
[c]Background absorbance originated from NADH/PPi/phosphate originally present in the sample
[d]Absorbance generated after reaction (b-c).
[e]Concentration was calculated using their standard curves.
[f]NADH/PPi/phosphate concentration (e) multiplied by the total volume of reaction mixture and divided by the reaction time.
[g]Enzyme concentration obtained through Bradford assay.
[h]Specific activity is obtained through (f) divided by (g).

Supplementary Table 2. Performance of EC number prediction tools for the Price-149 dataset.

Tool	Number of predicted sequences	Precision	Recall	F_1 score
CLEAN	149	0.5844	0.4671	0.4947
BLASTp	133	0.3717	0.2763	0.2969
HDMLF	135	0.1850	0.1447	0.1534
ProteInfer	55	0.2730	0.1447	0.1758
DeepECtransformer	115	0.5263	0.3026	0.3511

Supplementary Table 3. Performance of EC number prediction tools for the NEW-392 dataset.

Tool	Number of predicted sequences	Precision	Recall	F_1 score
CLEAN	392	0.5955	0.4791	0.4967
ProteInfer	205	0.4440	0.3201	0.3341
DeepECtransformer	363	0.4268	0.3260	0.3350

Supplementary Table 4. Performance of EC number prediction tools for the SwissProt2023 dataset.

Tool	Number of predicted sequences	Precision	Recall	F_1 score
CLEAN	1239	0.2683	0.2639	0.2530
DeepECtransformer	745	0.4504	0.2611	0.2889

Supplementary Table 5. Strains, plasmids, and primers used in this study.

Strain or plasmid	Description[a]	Source
M. succiniciproducens PALK	*M. succiniciproducens* (*ldhA*::Km^r *pta-ackA*::Sp^r)	Ref [2]
M. succiniciproducens PALK (pMS3-P93052)	PALK strain harboring pMS3-P93052	This study
E. coli BL21 (DE3) (pET22b(+)-ygfF)	BL21 (DE3) strain harboring pET22b(+)-ygfF	This study
E. coli BL21 (DE3) (pET22b(+)-yciO)	BL21 (DE3) strain harboring pET22b(+)-yciO	This study
E. coli BL21 (DE3) (pET22b(+)-yjdM)	BL21 (DE3) strain harboring pET22b(+)-yjdM	This study
E. coli TOP10	Str^r, Cloning host	Lab stock
pMS3	Ap^r, *E. coli*-rumen bacteria shuttle vector containing *M. succiniciproducens frdA* promoter (4.3 kb)	Ref [7]
pET22b(+)	Ap^r, plasmid containing his-tag and T7 promoter (5.4 kb)	Lab stock
pMS3-P93052	pMS3 derivative containing *P93052* gene from *Botryococcus braunii* (5.2 kb)	This study
pET22b(+)-ygfF	pET22b(+) derivative containing *ygfF* gene from *E. coli* (6.1 kb)	This study
pET22b(+)-yciO	pET22b(+) derivative containing *yciO* gene from *E. coli* (5.9 kb)	This study
pET22b(+)-yjdM	pET22b(+) derivative containing *yjdM* gene from *E. coli* (5.6 kb)	This study
**Primer**	**Sequence (5' to 3')**	
P1	TATCAACTCTACTGGGGAGGATGGCTCGCAAGAAGTACGC	
P2	TCTAGAGGATCCCCGGGTACTCAGGCCAGCGACTCGTC	
P3	AGTGGTGGTGGTGGTGGTGTTTCCCGCCCGCCAAATC	
P4	CTTTAAGAAGGAGATATACAATGGCTATAGCACTTGTGACTGG	
P5	AGTGGTGGTGGTGGTGGTGCTAAGAAAGGCTTCACATC	
P6	CTTTAAGAAGGAGATATACAATGAGCCAGTTTTTTATATTC	
P7	AGTGGTGGTGGTGGTGGTGGTTCTTTTTCACAAACTCAG	
P8	CTTTAAGAAGGAGATATACAATGTCATTACCACACTGC	

[a]Ap, ampicillin; Km, kanamycin; Sp, spectinomycin; Str, streptomycin; ^{r}, resistance.

Supplementary References

1. Lee, S.J., Song, H. & Lee, S.Y. Genome-based metabolic engineering of *Mannheimia succiniciproducens* for succinic acid production. *Appl. Environ. Microbiol.* **72**, 1939-1948 (2006).
2. Choi, S. et al. Highly selective production of succinic acid by metabolically engineered *Mannheimia succiniciproducens* and its efficient purification. *Biotechnol. Bioeng.* **113**, 2168-2177 (2016).
3. Ahn, J.H., Lee, J.A., Bang, J. & Lee, S.Y. Membrane engineering via trans-unsaturated fatty acids production improves succinic acid production in *Mannheimia succiniciproducens*. *J. Ind. Microbiol. Biotechnol.* **45**, 555-566 (2018).
4. Ahn, J.H. et al. Enhanced succinic acid production by *Mannheimia* employing optimal malate dehydrogenase. *Nat. Commun.* **11**, 1970 (2020).
5. Hua, Y.H., Wu, C.Y., Sargsyan, K. & Lim, C. Sequence-motif detection of NAD(P)-binding proteins: discovery of a unique antibacterial drug target. *Sci. Rep.* **4**, 6471 (2014).
6. Yu, T. et al. Enzyme function prediction using contrastive learning. *Science* **379**, 1358-1363 (2023).
7. Jang, Y.S. et al. Construction and characterization of shuttle vectors for succinic acid-producing rumen bacteria. *Appl. Environ. Microbiol.* **73**, 5411-5420 (2007).
