# Supplementary material for: Functional annotation of enzyme-encoding genes using deep learning with transformer layers
Source: Nat Commun. 2023 Nov 14;14:7370. doi: 10.1038/s41467-023-43216-z (PMC10645960; doi:10.1038/s41467-023-43216-z)
Supplement: Supplementary file 3 — Description of Additional Supplementary Files [file 41467_2023_43216_MOESM3_ESM.pdf]

Description of Additional Supplementary Files:

Supplementary Data 1: Predicted EC numbers for amino acid sequences from Swiss-Prot using DeepECtransformer.

Supplementary Data 2: Visualization of the latent representations of the amino acid sequences of enzymes in the Swiss-Prot database using TMAP.

Supplementary Data 3: Commonly highlighted motifs for each EC number using DeepECtransformer neural network. The data is available at doi.org/10.5281/zenodo.10023678.

Supplementary Data 4: Sequences for each of strain specific alleles.

Supplementary Data 5: EC number prediction results for the y-ome proteins.

Supplementary Data 6: EC numbers of 128,100,490 amino acid sequences in 70,600 genomes in NCBI. The data is available at doi.org/10.5281/zenodo.10023678.

Supplementary Data 7: Solubility prediction results for 295 y-ome proteins.
