# Supplementary material for: Functional annotation of enzyme-encoding genes using deep learning with transformer layers
Source: Nat Commun. 2023 Nov 14;14:7370. doi: 10.1038/s41467-023-43216-z (PMC10645960; doi:10.1038/s41467-023-43216-z)
Supplement: Supplementary file 11 — Reporting Summary [file 41467_2023_43216_MOESM11_ESM.pdf]

nature portfolio

Corresponding author(s): Sang Yup Lee

Last updated by author(s): Oct 20, 2023

Reporting Summary

Nature Portfolio wishes to improve the reproducibility of the work that we publish. This form provides structure for consistency and transparency in reporting. For further information on Nature Portfolio policies, see our Editorial Policies and the Editorial Policy Checklist.

Statistics

For all statistical analyses, confirm that the following items are present in the figure legend, table legend, main text, or Methods section.

n/a	Confirmed	
☐	☒	The exact sample size (n) for each experimental group/condition, given as a discrete number and unit of measurement
☐	☒	A statement on whether measurements were taken from distinct samples or whether the same sample was measured repeatedly
☐	☒	The statistical test(s) used AND whether they are one- or two-sided<br>*Only common tests should be described solely by name; describe more complex techniques in the Methods section.*
☒	☐	A description of all covariates tested
☐	☒	A description of any assumptions or corrections, such as tests of normality and adjustment for multiple comparisons
☐	☒	A full description of the statistical parameters including central tendency (e.g. means) or other basic estimates (e.g. regression coefficient) AND variation (e.g. standard deviation) or associated estimates of uncertainty (e.g. confidence intervals)
☐	☒	For null hypothesis testing, the test statistic (e.g. F, t, r) with confidence intervals, effect sizes, degrees of freedom and P value noted<br>*Give P values as exact values whenever suitable.*
☒	☐	For Bayesian analysis, information on the choice of priors and Markov chain Monte Carlo settings
☒	☐	For hierarchical and complex designs, identification of the appropriate level for tests and full reporting of outcomes
☒	☐	Estimates of effect sizes (e.g. Cohen's d, Pearson's r), indicating how they were calculated

Our web collection on statistics for biologists contains articles on many of the points above.

Software and code

Policy information about availability of computer code

Data collection	Biopython 1.78, NumPy 1.17.3, pandas 0.25.2, Python 3.6 were used.
Data analysis	Biopython 1.78, CD-hit package 4.6, Clustal Omega 1.2.3, DIAMOND 2.0.11, faerun 0.3.20, iTOL, Logomaker 0.8, matplotlib 3.2.2, MMSeq2, NumPy 1.17.3, pandas 0.25.2, Python 3.6, PyTorch 1.7.0, scikit-learn 0.21.3, tmap 1.0.4, transformers 3.5.1 (huggingface) were used.<br><br>The source code of this study is also available at GitHub (https://github.com/kaistsystemsbiology/DeepProZyme).

For manuscripts utilizing custom algorithms or software that are central to the research but not yet described in published literature, software must be made available to editors and reviewers. We strongly encourage code deposition in a community repository (e.g. GitHub). See the Nature Portfolio guidelines for submitting code & software for further information.

Data

Policy information about availability of data

All manuscripts must include a data availability statement. This statement should provide the following information, where applicable:

- Accession codes, unique identifiers, or web links for publicly available datasets
- A description of any restrictions on data availability
- For clinical datasets or third party data, please ensure that the statement adheres to our policy

Supplementary Data 1-7 are also available at doi.org/10.5281/zenodo.10023678 (ref. 52). Source data are provided with this paper and also available from Figshare:

https://doi.org/10.6084/m9.figshare.23577036 (ref. 51). UniProtKB/Swiss-Prot and TrEMBL entries (released in April 2018) were used to construct the uniprot dataset. Protein 3D structures used in this paper can be accessed with PDB ID 1IB6 [https://www.rcsb.org/structure/1IB6] and 1CIV [https://www.rcsb.org/structure/1CIV].

Research involving human participants, their data, or biological material

Policy information about studies with human participants or human data. See also policy information about sex, gender (identity/presentation), and sexual orientation and race, ethnicity and racism.

Reporting on sex and gender	None
Reporting on race, ethnicity, or other socially relevant groupings	None
Population characteristics	None
Recruitment	None
Ethics oversight	None

Note that full information on the approval of the study protocol must also be provided in the manuscript.

Field-specific reporting

Please select the one below that is the best fit for your research. If you are not sure, read the appropriate sections before making your selection.

[x] Life sciences [ ] Behavioural & social sciences [ ] Ecological, evolutionary & environmental sciences

For a reference copy of the document with all sections, see nature.com/documents/nr-reporting-summary-flat.pdf

Life sciences study design

All studies must disclose on these points even when the disclosure is negative.

Sample size	All sample sizes used are listed in the manuscript. No sample size calculation was performed. Most experiments were done in triplicates, which is a normally employed sample size to ensure biological reproducibility, unless otherwise specified. For bioreactor culture, experiments were conducted in duplicates as highly reproducible and small differences in values would not impact conclusions. Reference papers for the determination of biological sample sizes are as follows: PMID 37095132; PMID 31209347.
Data exclusions	No data were excluded from the analyses.
Replication	Experimental findings were reproduced by duplicates or triplicates.
Randomization	Single colonies were randomly selected from plates, each of which was subjected to independent fed-batch fermentations and chemical analyses. As colonies with similar size and apperance were randomly chosen, experimental group allocation was performed randomly.
Blinding	The investigators were blinded to the group allocation by randomly selecting single colonies multiple times.

Reporting for specific materials, systems and methods

We require information from authors about some types of materials, experimental systems and methods used in many studies. Here, indicate whether each material, system or method listed is relevant to your study. If you are not sure if a list item applies to your research, read the appropriate section before selecting a response.

Materials & experimental systems

n/a	Involved in the study	
[x]	[ ]	Antibodies
[x]	[ ]	Eukaryotic cell lines
[x]	[ ]	Palaeontology and archaeology
[x]	[ ]	Animals and other organisms
[x]	[ ]	Clinical data
[x]	[ ]	Dual use research of concern
[x]	[ ]	Plants

Methods

n/a	Involved in the study	
[x]	[ ]	ChIP-seq
[x]	[ ]	Flow cytometry
[x]	[ ]	MRI-based neuroimaging
